# Association between periodontal disease status and risk of atrial fibrillation: a nationwide population-based cohort study

**DOI:** 10.1186/s12903-023-03165-x

**Published:** 2023-07-08

**Authors:** Jung-Hyun Park, Hyungwoo Lee, Jin-Woo Kim, Tae-Jin Song

**Affiliations:** 1 Department of Oral and Maxillofacial Surgery, Mokdong Hospital, Ewha Womans University College of Medicine, Anyangcheon-Ro 1071, Yangcheon-Gu, Seoul, 07985 Republic of Korea; 2 Department of Neurology, Seoul Hospital, Ewha Womans University College of Medicine, 260, Gonghang-Daero, Gangseo-Gu, Seoul, 07804 Republic of Korea

**Keywords:** Periodontal disease, Atrial fibrillation, Oral health, Periodontitis

## Abstract

**Background:**

Periodontal disease can activate an immune process linked to systemic diseases, including atrial fibrillation. However, the relationship between periodontal disease and atrial fibrillation remains unclear.

**Aim:**

This study aimed to investigate whether changes in periodontal disease status are associated with the risk of atrial fibrillation.

**Methods:**

Using the National Health Insurance Database Korea, participants who underwent the first oral health examination in 2003 and a second examination in 2005–2006 without a history of atrial fibrillation were included. Participants were grouped according to changes in periodontal disease status during two oral examinations: periodontal disease-free, periodontal disease-recovered, periodontal disease-developed, and periodontal disease-chronic. The outcome was the incidence of atrial fibrillation.

**Results:**

The study included 1,254,515 participants, with a median follow-up of 14.3 years and 25,402 (2.02%) cases of atrial fibrillation occurred. During follow-up, the risk of atrial fibrillation was highest in the periodontal disease-chronic group, followed by the periodontal disease-developed, periodontal disease-recovered, and periodontal disease-free groups (p for trend < 0.001). Moreover, recovery from periodontal disease was associated with a reduced risk of atrial fibrillation compared to a chronic periodontal disease status (Hazard ratio: 0.97, 95% Confidence interval: 0.94—0.99, *p* = 0.045). The development of periodontal disease was associated with an increased risk of atrial fibrillation compared to being periodontal disease-free (Hazard ratio: 1.04, 95% Confidence interval: 1.01—1.08, *p* = 0.035).

**Conclusion:**

Our findings suggest that changes in periodontal disease status impact the risk of atrial fibrillation. Management of periodontal disease may help prevent atrial fibrillation.

**Supplementary Information:**

The online version contains supplementary material available at 10.1186/s12903-023-03165-x.

## Introduction

Atrial fibrillation (AF) is one of the most common cardiac arrhythmia worldwide and a major risk factor for systemic thromboembolism and stroke [1–5]. Previous studies have also shown that AF leads to critical morbidity and mortality [6, 7]. With the global population aging, the disease burden of AF is expected to increase, [4, 8, 9] making risk factor identification in AF crucial. Factors such as hypertension, cardiomyopathy, smoking, and alcohol consumption showed its association with AF to date [10]. Further research, especially in finding the modifiable risk factors for developing AF, is one of the major concerns.

Periodontal disease is a serious gum infection that damages the surrounding soft tissues and can lead to destruction of the bony structure that supports teeth without treatment. Moreover, periodontal disease can also provoke transient bacteremia and systemic inflammation, linked to the development of systemic diseases, including AF [11–14]. Studies have reported that periodontal disease is associated with increased levels of systemic inflammatory biomarkers and correlated with various cardiovascular disorders, including coronary artery disease and AF [15, 16]. Numerous studies conducted on both animals and humans suggest that systemic inflammation is a significant factor in the development of atrial remodeling, which serves as the initial substrate for the onset of AF [16, 17]. Although periodontal disease is highly prevalent in the general population, it is readily treatable and preventable [18]. Therefore, periodontal disease could be a significant modifiable risk factor in the development of AF. However, current research lacks a large sample size study examining the correlation between the persistence or improvement of periodontal disease and AF risk.

In this study, we aimed to investigate the association between changes in periodontal disease status and the risk of developing AF in a nationwide general population based on a longitudinal study. We hypothesized that the risk of developing AF may vary based on whether periodontal disease improves or persists over time.

## Methods

### Database

The National Health Insurance Database, provided by the Korean National Health Insurance Service (NHIS), was used in this study. The database contains medical information of the entire population of Korea with demographics, socioeconomic status, type of health insurance coverage, and nation-supported health examination information [19]. In Korea, the NHIS is the sole insurance provider. It is controlled and supported by the Korean government. The NHIS covers approximately 97% of the Korean population; the Medical Aid program of NHIS supports the remaining 3%. Subscribers of the NHIS are recommended to receive standardized medical health examinations every 1–2 years [20]. While the primary goal of health examinations is to enhance the health of the entire population by detecting diseases early and offering guidance on disease management, the NHIS anonymizes the collected data from participants and offers it as a research database to health researchers for research purposes. We requested data from NHIS on individuals over 20 years who underwent oral health examinations in 2003 (dataset number: NIHS-2022–01-313). The data contains demographic information, medical information—the claims database of diagnosis, treatment, and prescription, and health examination information—height, weight, household income, health-related lifestyle, and oral health status examined by dentists. This study was approved by the Institutional Review Board of Ewha Womans University College of Medicine (2021–07–034) and received a consent waiver.

### Study population and variables

**Fig. 1:**
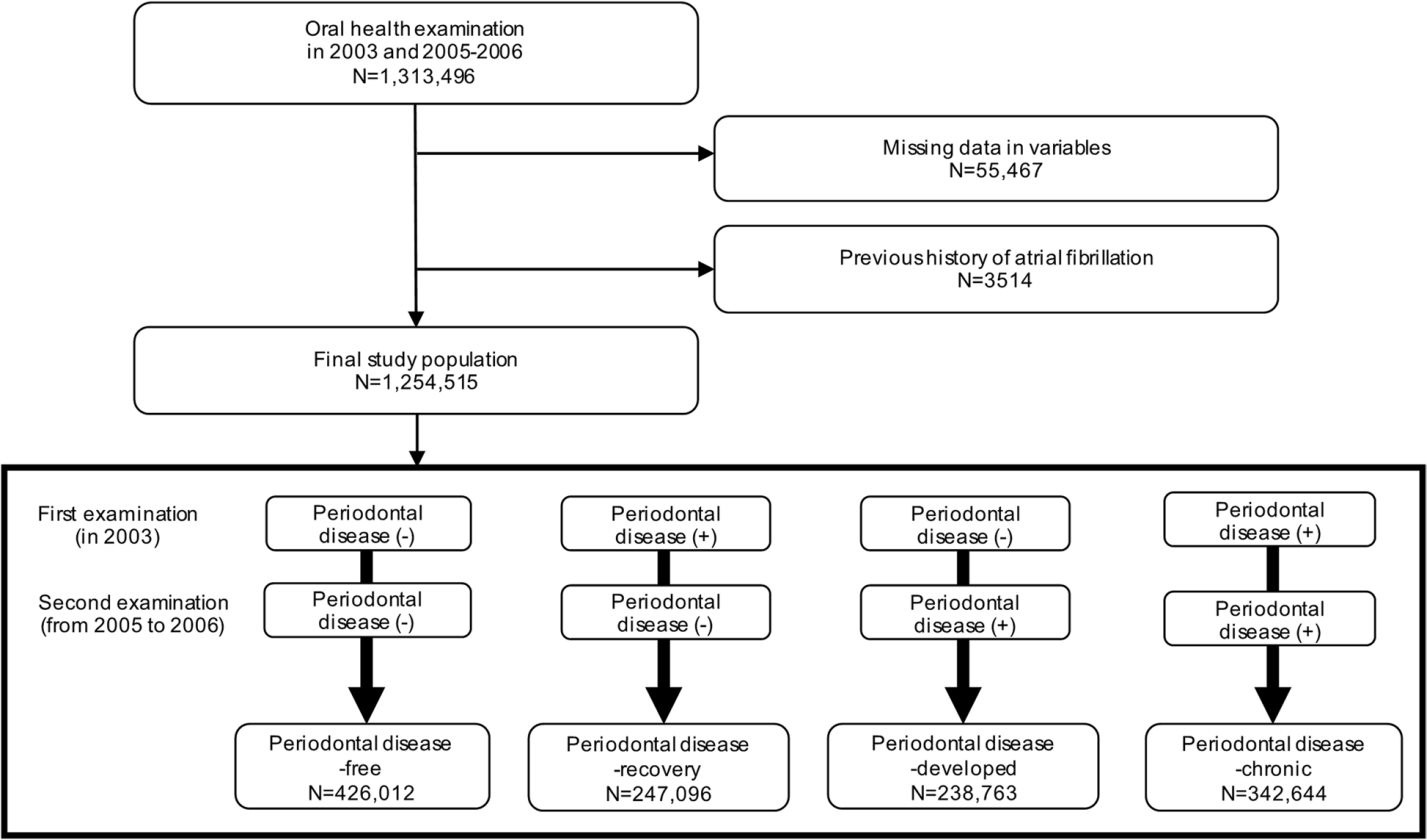
Flowchart of study participant selection

This study included all participants (*n* = 1,313,496) who underwent two consecutive oral health examinations during the first period (in 2003) and the second period (from 2005 to 2006). We excluded participants with missing data for variables of interest (*n* = 55,467) and a previous history of AF (*n* = 3,514). Finally, the study included 1,254,515 participants for analysis (Fig. 1).

The participants received two oral examinations: the first one in 2003 and the second one in 2005–2006. Professional dentists, who completed training prior to the examinations, assessed periodontal disease during these examinations. Study participants were divided into four groups based on the changes in periodontal disease status at the two oral examinations. Periodontal disease was evaluated by measurement of the periodontal pocket depth, a widely used indicator of periodontal tissue destruction, or by assessing gingival inflammation during oral health examination [21–23]. The evaluation of gingival inflammation involves visual assessment of changes in gingival color, loss of stippling, and bleeding upon probing. The four groups are as follows: (1) periodontal disease-free (participants who were consistently free from periodontal disease during the two examinations), (2) periodontal disease-recovered (participants who had periodontal disease at the first examination but were free at the second examination), (3) periodontal disease-developed (participants without periodontal disease at the first examination but newly developed periodontal disease at the second examination), and (4) periodontal disease-chronic (participants who had periodontal disease at both examinations) [21, 24].

The occurrence of AF after the second oral health examination was monitored to investigate the association between changes in periodontal disease status and the risk of developing AF longitudinally. The index date was the date of the second oral health examination. The outcome was the occurrence of AF (International Classification of Diseases (ICD)-10 code: I48) with at least two claims per year. These codes were validated and/or used from a previous study [25]. The diagnostic accuracy of AF was validated by reviewing electrocardiograms, and the positive predictive value for diagnosis was 94.1% [24, 25].

Regarding covariates, the following characteristics and variables were collected at the index date: age, sex, body mass index, and household income. Details of smoking status, alcohol consumption (days per week), and regular physical exercise (frequency per week) were obtained using self-questionnaires. Smoking status was divided into three categories: none, former smoker, and current smoker. The definition of comorbidities and the Charlson Comorbidity Index is described in Supplementary methods [13, 26–31].

### Statistical analysis

The baseline characteristics between groups were compared using the Chi-square test for categorical variables and analysis of variance for continuous variables with Bonferroni post-hoc analysis. Continuous variables are presented as mean ± standard deviation, and categorical variables are presented as numbers (percentages). Kaplan–Meier survival curves with the log-rank test were used to evaluate the relationship between changes in periodontal disease status and the occurrence of AF. Cox proportional hazard regression was used with adjustment for confounding variables to determine the hazard ratio (HR). In multivariable Cox regression, age, sex, body mass index, household income, smoking status, alcohol consumption, physical activity, comorbidities, and the Charlson Comorbidity Index were adjusted. The results of Cox regression analysis were described as HR and 95% confidence interval (CI). The assumption of the proportionality of hazards was tested using Schoenfeld residuals. The model showed no deviation from the proportional hazards assumption. Pairwise comparison analysis was performed to assess the altered AF risk for those who recovered from or developed periodontal disease, periodontal disease-recovered vs. periodontal disease-free, periodontal disease-developed vs. periodontal disease-free, periodontal disease-recovered vs. periodontal disease-chronic, and periodontal disease-developed vs. periodontal disease-chronic. For sensitivity analysis, a multivariable analysis was performed after excluding participants with AF within one year from the index date to minimize the possibility of reverse causality (landmark analysis). All statistical analyses were performed using Statistical Analysis System software (SAS version 9.2, SAS Institute, Cary, NC). All values with *p*-values < 0.05 were considered statistically significant.

## Results

All included participants, 426,012 (34.0%), 247,096 (19.7%), 238,763 (19.0%), and 342,644 (27.3%), were categorized into periodontal disease-free, periodontal disease-recovered, periodontal disease-developed, and periodontal disease-chronic groups, respectively. The median interval between the first and second oral health examination was 21.5 months (interquartile range, 11.1–25.5 months). Baseline characteristics according to changes in periodontal disease status are presented in Table 1. The mean age of overall participants was 42.09 ± 11.00 years, and 72.9% were male. In the periodontal disease-chronic group, the proportion of men was higher than in other groups; the periodontal disease-free group had a higher proportion of women than other groups.

**Table 1:** Baseline characteristics of the study population

	**Total**	**Periodontal disease-free**	**Periodontal disease-recovered**	**Periodontal disease-developed**	**Periodontal disease-chronic**	***p*** **-value**
**Number of patients**	1,254,515	426,012 (34.0)	247,096 (19.7)	238,763 (19.0)	342,644 (27.3)	
**Age, years**	42.09 ± 11.00	41.26 ± 11.27	42.06 ± 11.04	42.09 ± 10.71	43.16 ± 10.73	< 0.001
**Sex**						< 0.001
Men	914,065 (72.9)	281,317 (66.0)	180,499 (73.0)	179,180 (75.0)	273,069 (79.7)	
Women	340,450 (27.1)	144,695 (34.0)	66,597 (27.0)	59,583 (25.0)	69,575 (20.3)	
**BMI (kg/m**^**2**^**)**	23.58 ± 3.43	23.29 ± 3.00	23.62 ± 3.03	23.66 ± 4.74	23.86 ± 3.04	< 0.001
**Household income**						< 0.001
Q1, lowest	152,522 (12.2)	52,299 (12.3)	29,858 (12.1)	27,422 (11.5)	42,943 (12.5)	
Q2	448,653 (35.8)	152,663 (35.8)	88,128 (35.7)	82,123 (34.4)	125,739 (36.7)	
Q3	447,669 (35.7)	150,910 (35.4)	88,923 (36.0)	87,367 (36.6)	120,469 (35.2)	
Q4, highest	205,671 (16.4)	70,140 (16.5)	40,187 (16.3)	41,851 (17.5)	53,493 (15.6)	
**Smoking status**						< 0.001
None	680,345 (54.2)	263,493 (61.9)	135,205 (54.8)	124,659 (52.2)	156,988 (45.8)	
Former	174,387 (13.9)	60,235 (14.1)	34,675 (14.0)	34,107 (14.3)	45,370 (13.2)	
Current	399,783 (31.9)	102,284 (24.0)	77,216 (31.3)	79,997 (33.5)	140,286 (40.9)	
**Alcohol consumption (days/week)**						< 0.001
< 1	4190 (0.3)	1210 (0.3)	870 (0.4)	791 (0.3)	1319 (0.4)	
1–2	817,263 (65.2)	296,397 (69.6)	161,909 (65.5)	152,666 (63.9)	206,291 (60.2)	
≥ 3	433,062 (34.5)	128,405 (30.1)	84,317 (34.1)	85,306 (35.7)	135,034 (39.4)	
**Regular physical activity (days/week)**						< 0.001
< 3	1,015,033 (80.9)	342,441 (80.4)	199,004 (80.5)	193,334 (81.0)	280,254 (81.8)	
≥ 3	239,482 (19.1)	83,571 (19.6)	48,092 (19.5)	45,429 (19.0)	62,390 (18.2)	
**Comorbidities**						
Hypertension	549,267 (43.8)	173,473 (40.7)	107,227 (43.4)	106,625 (44.7)	161,942 (47.3)	< 0.001
Diabetes mellitus	163,402 (13.0)	51,584 (12.1)	31,312 (12.7)	31,679 (13.3)	48,827 (14.3)	< 0.001
Dyslipidemia	288,542 (23.0)	92,676 (21.8)	57,918 (23.4)	55,115 (23.1)	82,833 (24.2)	< 0.001
Cancer	20,024 (1.6)	7243 (1.7)	4065 (1.7)	3775 (1.6)	4941 (1.4)	< 0.001
Renal disease	10,766 (0.9)	3436 (0.8)	2157 (0.9)	2041 (0.9)	3132 (0.9)	< 0.001
**Charlson Comorbidity Index**						< 0.001
0	534,029 (42.5)	176,372 (41.4)	104,765 (42.4)	101,024 (42.3)	151,868 (44.3)	
1	504,294 (40.2)	175,999 (41.3)	99,303 (40.2)	96,657 (40.5)	132,335 (38.6)	
≥ 2	216,192 (17.2)	73,641 (17.3)	43,028 (17.4)	41,082 (17.2)	58,441 (17.1)	

Data are presented as mean ± standard deviation or number (percentage)

*BMI* Body mass index, *Q* Quartile

The frequency of alcohol consumption was highest in the periodontal disease-chronic group, followed by the periodontal disease-developed and periodontal disease-recovered groups, and was lowest in the periodontal disease-free group. A similar trend was observed for smoking status. Regarding comorbidities, the prevalence of hypertension, diabetes mellitus, dyslipidemia, and renal disease was lowest in the periodontal disease-free group and increased among participants in the periodontal disease-recovered and periodontal disease-developed groups, being highest in the periodontal disease-chronic group. In contrast, the frequency of cancer and Charlson Comorbidity Index ≥ 2 were highest among those in the periodontal disease-free group compared to other groups (Table 1).

**Fig. 2:**
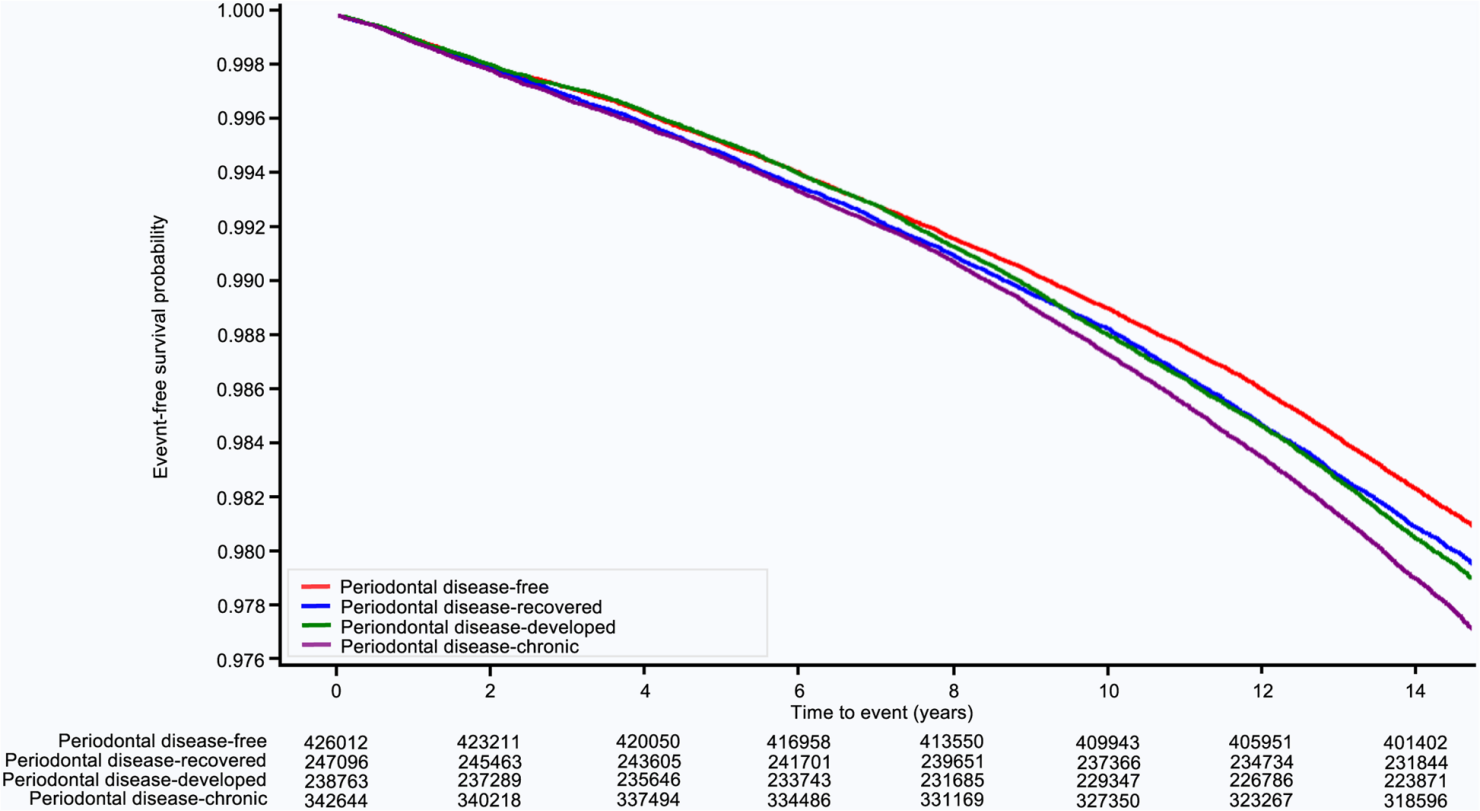
Kaplan–Meier survival curves for atrial fibrillation occurrence according to periodontal disease status

During a median follow-up of 14.3 years, 25,402 (2.02%) cases of AF occurred. The Kaplan–Meier survival curves for the occurrence of AF according to changes in periodontal disease status are shown in Fig. 2. The participants had an altered risk of AF according to changes in periodontal disease status. The risk of occurrence of AF was highest in the periodontal disease-chronic group throughout follow-up, followed by the periodontal disease-developed group, periodontal disease-recovered group, and periodontal disease-free group (p for trend < 0.001) (Table 2). In the multivariate analysis, the risk of AF occurrence was not significantly different between the periodontal disease-recovered group and the periodontal disease-free group (HR: 1.00, 95% CI: 0.97 – 1.04, *p* = 0.998). However, the risk of AF was higher in both the periodontal disease-developed (HR: 1.03, 95% CI: 1.01—1.06, *p* = 0.041) and periodontal disease-chronic groups (HR: 1.04, 95% CI: 1.01—1.07, *p* = 0.019) compared to the periodontal disease-free group (p for trend = 0.032) (Table 2, Supplementary Table 1).

**Table 2:** The association between change in periodontal disease status and incident atrial fibrillation risk

**Group**	**Event rate (%)**	**Person-years**	**Incidence rate** **(per 1000 person-years)**	**Unadjusted**			**Adjusted** ^a^		
				**HR (95% CI)**	***p*** **-value**	**p for trend**	**HR (95% CI)**	***p*** **-value**	**p for trend**
**Periodontal disease-free**	1.87	6,098,095.73	1.30	1 (reference)		< 0.001	1 (reference)		0.032
**Periodontal disease-recovered**	1.99	3,537,490.38	1.39	1.07 (1.03,1.11)	< 0.001		1.00 (0.97,1.04)	0.998	
**Periodontal disease-developed**	2.05	3,414,135.87	1.43	1.10 (1.06,1.14)	< 0.001		1.03 (1.01,1.06)	0.041	
**Periodontal disease-chronic**	2.23	4,892,083.45	1.56	1.20 (1.16,1.23)	< 0.001		1.04 (1.01,1.07)	0.019	

*HR* Hazard ratio, *CI* Confidence interval

^a^Adjusted for age, sex, body mass index, household income, smoking status, alcohol consumption, regular physical activity, hypertension, diabetes mellitus, dyslipidemia, cancer, renal disease, and Charlson Comorbidity Index

In a further pairwise comparison, the periodontal disease-recovered group had a relatively lower risk of AF than the periodontal disease-chronic group (HR: 0.97, 95% CI: 0.94—0.99, *p* = 0.045). The periodontal disease-developed group demonstrated a higher risk of AF than the periodontal disease-free group (HR: 1.04, 95% CI: 1.01—1.08, *p* = 0.035) in multivariable analysis (Table 3, Supplementary Table 2). Sensitivity analysis consistently confirmed the association between changes in periodontal disease status and the risk of incident AF (Supplementary Table 3).

**Table 3:** Pairwise comparisons of the association between change in periodontal disease status and risk of incident atrial fibrillation

	**Unadjusted**		**Adjusted** ^a^	
	**HR (95% CI)**	***p*** **-value**	**HR (95% CI)**	***p*** **-value**
**Periodontal disease -recovered vs. Periodontal disease-free (reference)**	1.07 (1.03,1.10)	< 0.001	0.99 (0.96,1.03)	0.646
**Periodontal disease-developed vs. Periodontal disease-free (reference)**	1.10 (1.06,1.10)	< 0.001	1.04 (1.01,1.08)	0.035
**Periodontal disease-recovered vs. Periodontal disease-chronic (reference)**	0.89 (0.86,0.93)	< 0.001	0.97 (0.94,0.99)	0.045
**Periodontal disease-developed vs. Periodontal disease-chronic (reference)**	0.92 (0.89,0.96)	< 0.001	1.00 (0.96,1.03)	0.906

*HR* Hazard ratio, *CI* Confidence interval

^a^Adjusted for age, sex, body mass index, household income, smoking status, alcohol consumption, regular physical activity, hypertension, diabetes mellitus, dyslipidemia, cancer, renal disease, and Charlson Comorbidity Index

## Discussion

The main finding of this study is that changes in periodontal disease status are associated with changes in the risk of AF. The risk of AF occurrence was highest in individuals with persistent periodontal disease. Moreover, compared to individuals with persistent periodontal disease, those who recovered from it had a lower risk for AF occurrence. Furthermore, those who developed periodontal disease had a higher risk of AF than those who remained free from periodontal disease.

Several previous studies have reported an association between periodontal disease and AF. Study in dogs with periodontitis have shown that the inflammatory response, both locally in the atrial myocardium and systemically, increases the susceptibility to AF [32]. In a population-based cohort study using the Taiwanese National Health Insurance Research Database, patients with periodontal disease had an increased risk of atrial fibrillation [33]. A recent study using the Atherosclerosis Risk in Communities Study cohort found that individuals with severe periodontal disease had a higher incidence of AF than those with healthy periodontal status. Moreover, regular dental care users had a lower risk of incident AF than those who received episodic dental care [34]. However, previous studies only considered the risk of AF by examining the presence or absence of periodontal disease at a single time point, disregarding the impact of changes in periodontal disease, which can be treated and managed. There is limited evidence from population-based studies investigating whether recovery from or development of periodontal disease is associated with an altered risk for AF. The significance of the study is that it provides evidence for the association between changes in periodontal disease status and changes in the risk of AF, based on a large population-based study that used a series of oral examinations. This study provides a comprehensive understanding of how changes in periodontal disease status can impact the risk of AF. The findings may suggest that recovery from periodontal disease may lower the risk of AF, while the development of periodontal disease may increase the risk of AF.

Although the exact mechanism linking periodontal disease and AF remains unclear, there are several potential explanations for the association. First, subgingival biofilm in periodontal pockets can allow oral bacteria to enter the systemic circulation, leading to bacteremia [35]. *Porphyronomas gingivalis*, one of the key bacteria associated with periodontal disease, along with its virulence factors and inflammatory mediators, may contribute to cardiac remodeling and the development of AF [36]. Second, systemic inflammation resulting from periodontal disease may also be linked to AF. Previous studies have shown that elevated levels of serum inflammatory markers, such as tumor necrosis factor-α, interleukin-6, interleukin-2, and C-reactive protein, are associated with an increased risk of AF and are commonly found in patients with periodontal disease [37, 38]. These pro-inflammatory conditions could promote AF occurrence through electrical and structural remodeling. Conversely, it seems that recovering from periodontal disease can lower the chances of developing AF by altering the oral microbiome and reducing inflammation. Since periodontal bacteria can be reduced through scaling or brushing, [39] regaining periodontal health can directly decrease the risk of AF caused by bacteremia. Furthermore, periodontal therapy has been shown to lower inflammatory markers, [40] so recovering from periodontal disease can potentially have AF preventive effects by decreasing the overall systemic inflammatory response. In this study, the risk of AF occurrence was highest in individuals who had persistent periodontal disease. Compared to those with persistent periodontal disease, individuals who recovered from periodontal disease had a reduced risk of AF occurrence. Additionally, those who developed periodontal disease had a higher risk of AF than those who remained periodontal disease-free. This suggests that periodontal disease may alter oral bacterial and inflammatory burden, which in turn alters the risk of AF occurrence.

This study has several limitations. First, prior periodontal treatment history was not considered when determining the study groups, which could be crucial, especially in individuals who recovered from periodontal disease. Further research is necessary to investigate the potential association between recovery from periodontal disease with periodontal treatment and reduced risk of AF. Second, data from NHIS did not provide information on the severity of periodontal disease, making it impossible to assess the risk of developing AF based on disease severity. Third, the study did not account for potential confounding factors affecting AF development, such as laboratory findings, which could impact the results. Fourth, as the study included only Koreans, the results may not be generalizable to other racial or ethnic groups. Fifth, variables of individual health habits could be biased as they were self-reported through questionnaires. Sixth, the participating dentists were required to complete training prior to the oral examinations, but the database used in the study does not provide detailed information about the calibration of the examiners and the intra and inter-examiner agreement. Seventh, the status of periodontal disease was not assessed at the time of atrial fibrillation diagnosis due to the retrospective nature of this longitudinal cohort study. This limitation should be taken into consideration when interpreting the results. Despite limitations, this study had the strength of analyzing the effects of alteration of periodontal disease on AF occurrence in a large cohort using a long-term follow-up. The results of this study offer significant evidence supporting the importance of recovering from and preventing periodontal disease in reducing the risk of AF.

## Conclusion

In conclusion, altered periodontal disease status is closely associated with altered risks for AF. The benefit of periodontal disease recovery, or harm associated with periodontal disease development, may present. Further studies to confirm the benefits of recovery from or prevention of periodontal disease are warranted to reduce the burden of AF.

## Supplementary Information

**Additional file 1.**

**Additional file 2.**

## Data Availability

The data used in this study are available in the National Health Insurance Service (NHIS) database; however, restrictions apply to the public availability of these data used under license for the current study. Requests for access to NHIS data can be made through the National Health Insurance Sharing Service homepage [http://nhiss.nhis.or.kr/bd/ab/bdaba021eng.do]. To access the database, a completed application form, research proposal, and application for approval from the institutional review board should be submitted to the inquiry committee of research support in the NHIS for review.

## Abbreviations

AF: Atrial fibrillation
NHIS: National Health Insurance Service
ICD: International Classification of Diseases
HR: Hazard ratio
CI: Confidence interval
