# Supplementary material for: Association between periodontal disease status and risk of atrial fibrillation: a nationwide population-based cohort study
Source: BMC Oral Health. 2023 Jul 8;23:461. doi: 10.1186/s12903-023-03165-x (PMC10329345; doi:10.1186/s12903-023-03165-x)
Supplement: Supplementary file 1 — Additional file 1. [file 12903_2023_3165_MOESM1_ESM.docx]

**Supplementary Methods**

Individual comorbidities between 2002 and the index date were identified. Diabetes mellitus was defined as satisfying one of these criteria: 1) at least one claim for relevant diagnostic codes (ICD-10 E11–14) with the prescription of an anti-diabetic agent, 2) two or more claims for relevant diagnostic codes (ICD-10 E11–14), 3) fasting serum glucose level ≥ 7.0 mmol/L, or 4) diabetes mellitus self-reported in the questionnaire. Dyslipidemia was defined as satisfying one of following criteria: 1) at least one claim for the relevant diagnostic code (ICD-10 E78) with the prescription of dyslipidemia-related agent, 2) two or more claims for the relevant diagnostic code (ICD-10 E78), or 3) total cholesterol ≥ 240 mg/dL. Atrial fibrillation was defined as two or more claims for the relevant diagnostic code (ICD-10 I48). Cancer was defined as having once admission or at least three outpatient claims for relevant diagnostic codes (ICD-10 C00–97) with specific registration code of ‘V027’ or ‘V193–4’. Renal disease was defined as two or more claims for relevant diagnostic codes (ICD-10 N17-19, I12-13, E082, E102, E112, E132) or an estimated glomerular filtration rate less than 60 mL/min/1.73 m^2^. The Charlson Comorbidity Index was defined as based on previous studies.^1-8^

**References**

**1.** Song TJ, Kim JW, Kim J. Oral health and changes in lipid profile: A nationwide cohort study. *Journal of clinical periodontology.* 2020;47:1437-1445.

**2.** Woo HG, Chang Y, Lee JS, Song TJ. Association of Tooth Loss with New-Onset Parkinson's Disease: A Nationwide Population-Based Cohort Study. *Parkinsons Dis.* 2020;2020:4760512.

**3.** Chang Y, Woo HG, Lee JS, Song TJ. Better oral hygiene is associated with lower risk of stroke. *J Periodontol.* 2021;92:87-94.

**4.** Lee K, Lee JS, Kim J, et al. Oral health and gastrointestinal cancer: A nationwide cohort study. *J Clin Periodontol.* 2020;47:796-808.

**5.** Kim J, Kim HJ, Jeon J, Song TJ. Association between oral health and cardiovascular outcomes in patients with hypertension: a nationwide cohort study. *J Hypertens.* 2022;40:374-381.

**6.** Song TJ, Chang Y, Jeon J, Kim J. Oral health and longitudinal changes in fasting glucose levels: A nationwide cohort study. *PLoS One.* 2021;16:e0253769.

**7.** Charlson ME, Carrozzino D, Guidi J, Patierno C. Charlson Comorbidity Index: A Critical Review of Clinimetric Properties. *Psychotherapy and Psychosomatics.* 2022;91:8-35.

**8.** Chang Y, Lee H, Song TJ. Association of gamma-glutamyl transferase variability with risk of venous thrombosis. Scientific reports. 2023;13:7402.
