# Supplementary material for: Association between periodontal disease status and risk of atrial fibrillation: a nationwide population-based cohort study
Source: BMC Oral Health. 2023 Jul 8;23:461. doi: 10.1186/s12903-023-03165-x (PMC10329345; doi:10.1186/s12903-023-03165-x)
Supplement: Supplementary file 2 — Additional file 2. [file 12903_2023_3165_MOESM2_ESM.docx]

SUPPLEMENTARY MATERIAL

# Supplementary Table 1. Results of multivariable analysis for occurrence of atrial fibrillation

| Group | Adjusted HR (95% CI) | p-value | p for trend |
| --- | --- | --- | --- |
| **Age, years** | 1.08 (1.08, 1.08) | <0.001 |  |
| **Sex** |  |  |  |
| Men | 1 (reference) |  |  |
| Women | 0.66 (0.64, 0.68) | <0.001 |  |
| **BMI (kg/m^2^)** | 1.00 (1.00, 1.01) | <0.001 |  |
| **Household income** |  |  | <0.001 |
| Q1, lowest | 1 (reference) |  |  |
| Q2 | 0.99 (0.94, 1.02) | 0.353 |  |
| Q3 | 1.03 (0.99, 1.07) | 0.187 |  |
| Q4, highest | 1.07 (1.03, 1.12) | 0.002 |  |
| **Smoking status** |  |  | <0.001 |
| None | 1 (reference) |  |  |
| Former | 1.08 (1.04, 1.12) | <0.001 |  |
| Current | 1.10 (1.06, 1.16) | <0.001 |  |
| **Alcohol consumption**  **(days/week)** |  |  | <0.001 |
| <1 | 1 (reference) |  |  |
| 1-2 | 0.93 (0.79, 1.09) | 0.378 |  |
| ≥3 | 1.05 (0.89, 1.23) | 0.593 |  |
| **Regular physical activity**  **(days/week)** |  |  |  |
| <3 | 1 (reference) |  |  |
| ≥3 | 0.94 (0.92, 0.97) | 0.008 |  |
| **Comorbidities** |  |  |  |
| Hypertension | 1.50 (1.46, 1.54) | <0.001 |  |
| Diabetes mellitus | 1.07 (1.03, 1.10) | <0.001 |  |
| Dyslipidemia | 1.01 (0.98, 1.03) | 0.646 |  |
| Cancer | 1.03 (0.96, 1.10) | 0.453 |  |
| Renal disease | 1.21 (1.11, 1.31) | <0.001 |  |
| **Charlson Comorbidity Index** |  |  | <0.001 |
| 0 | 1 (reference) |  |  |
| 1 | 1.25 (1.21, 1.29) | <0.001 |  |
| ≥2 | 1.53 (1.48, 1.59) | <0.001 |  |
| **Study group** |  |  | 0.032 |
| Periodontal disease-free | 1 (reference) |  |  |
| Periodontal disease-recovered | 1.00 (0.97, 1.04) | 0.998 |  |
| Periodontal disease-developed | 1.03 (1.01, 1.06) | 0.041 |  |
| Periodontal disease-chronic | 1.04 (1.01, 1.07) | 0.019 |  |

* Adjusted for age, sex, body mass index, household income, smoking status, alcohol consumption, regular physical activity, hypertension, diabetes mellitus, dyslipidemia, cancer, renal disease, and Charlson Comorbidity Index. HR, hazard ratio; CI, confidence interval;

# Supplementary Table 2. Results of pairwise comparison of the association between change in periodontal disease status and risk of incident atrial fibrillation

| Group1 | Group2 | p-value  (Raw) | p-value  (Bonferroni) |
| --- | --- | --- | --- |
| Periodontal disease-free | Periodontal disease-recovered | <0.001 | <0.001 |
| Periodontal disease-free | Periodontal disease-developed | <0.001 | <0.001 |
| Periodontal disease-free | Periodontal disease-chronic | <0.001 | <0.001 |
| Periodontal disease-recovered | Periodontal disease-developed | 0.138 | 0.825 |
| Periodontal disease-recovered | Periodontal disease-chronic | <0.001 | <0.001 |
| Periodontal disease-developed | Periodontal disease-chronic | <0.001 | <0.001 |

# Supplementary Table 3. The association between change in periodontal disease status and incident atrial fibrillation risk (landmark analysis)

| Group | Adjusted HR (95% CI) | p-value | p for trend |
| --- | --- | --- | --- |
|  |  |  | 0.016 |
| Periodontal disease-free | 1 (reference) |  |  |
| Periodontal disease-recovered | 1.00 (0.97, 1.04) | 0.872 |  |
| Periodontal disease-developed | 1.04 (1.01, 1.06) | 0.022 |  |
| Periodontal disease-chronic | 1.04 (1.01, 1.08) | 0.010 |  |

* Adjusted for age, sex, body mass index, household income, smoking status, alcohol consumption, regular physical activity, hypertension, diabetes mellitus, dyslipidemia, cancer, renal disease, and Charlson Comorbidity Index. HR, hazard ratio; CI, confidence interval;
